# Impact of the COVID-19 Pandemic on Clostridioides difficile Infection Rates in a Tertiary Hospital: A Retrospective Comparative Study

**DOI:** 10.7759/cureus.86045

**Published:** 2025-06-15

**Authors:** Haitham Shabana, Hesham Abdelwahed

**Affiliations:** 1 Department of Critical Care Medicine, Westmead Hospital, Sydney, AUS; 2 Department of Critical Care Medicine, Maitland Hospital, Newcastle, AUS

**Keywords:** antibiotic use, clostridioides difficile infection, covid-19, hand hygiene, icu

## Abstract

Introduction

The impact of the COVID-19 pandemic on the incidence of *Clostridioides difficile* infection (CDI) remains uncertain, with conflicting findings reported in the literature. While some studies have shown an increase in CDI cases, others have found no significant change. This study aimed to compare healthcare-associated CDI incidence rates (including community-onset and hospital-onset cases) between the pre-COVID-19 and COVID-19 periods at our institution, with secondary analyses of ICU and hospital length of stay, mortality, severity, and recurrence rates.

Methods

This single-center retrospective study at Wollongong Hospital compared CDI rates before and during the COVID-19 pandemic. Adult patients with diarrhea and confirmed CDI were included. Data on demographics, comorbidities, antibiotic use, hospital and ICU length of stay, mortality, and infection control metrics were analyzed. CDI incidence was calculated per 1,000 patient-days and per 1,000 admissions.

Results

Among 544 CDI cases (215 pre-COVID-19 and 329 during COVID-19), 291 (53.5%) were community-onset and 253 (46.5%) hospital-onset. There were no significant differences between the two periods in CDI type (p = 0.599), time to acquisition (p = 0.388), severity (17.2% vs. 19.5%), ICU admission (18.1% vs. 20.4%), ICU (p = 0.415) or hospital length of stay (p = 0.788), or mortality (9.3% vs. 8.8%). However, CDI recurrence was significantly higher during the COVID-19 period (10.9% vs. 5.6%, p < 0.05), as were incidence rates per 1,000 patient admissions (2.2 vs. 1.47, p < 0.001) and per 1,000 patient-days (0.64 vs. 0.45, p < 0.001). Hand hygiene compliance in general wards declined slightly but significantly (from 88.5% to 87.9%, p = 0.003), while ICU compliance remained stable.

Conclusion

The incidence of CDI was significantly higher during the COVID-19 period compared to the pre-COVID-19 period at our institution. These findings underscore the importance of strengthened antimicrobial stewardship and preventive measures during healthcare crises.

## Introduction

The Coronavirus Disease 2019 (COVID-19) pandemic severely challenged healthcare systems, prompting the creation of dedicated COVID-19 wards and Intensive Care Units (ICUs). These units operated under strict isolation protocols, with rigorous hand hygiene and personal protective equipment (PPE) used by healthcare workers [1].

*Clostridioides difficile* infection (CDI) is a common healthcare-associated infection in Australian hospitals, often triggered or exacerbated by excessive antibiotic use and suboptimal infection control practices [2]. The impact of the COVID-19 pandemic on CDI incidence remains uncertain. While some studies report an increase in CDI acquisition during the pandemic [3-7], others observed no significant change [8-10], or even a decline [11-18].

Proposed reasons for increased CDI acquisition during the pandemic include excessive antibiotic use, prolonged hospital stays, higher patient volumes, and older patients with more comorbidities [19,20]. Conversely, the decrease in CDI rates observed in some settings has been attributed to enhanced infection control measures such as contact precautions, PPE use, hand hygiene education, reduced hospital admissions, lower stool testing rates, and stricter transmission precautions [16,18,21].

This study aims to evaluate the impact of the COVID-19 pandemic on the incidence of CDI, both healthcare-associated community-onset and hospital-onset, by comparing CDI incidence rates (primary outcome), as well as ICU length of stay (LOS), hospital LOS, mortality, CDI severity, and CDI recurrence rates (secondary outcomes) between the pre-COVID-19 and COVID-19 periods at our institution.

## Materials and methods

Study design

This single-center, retrospective observational study was conducted at Wollongong Hospital, New South Wales, Australia. This study was approved by the Illawarra Shoalhaven Local Health District (ISLHD) review board on March 8, 2024 (Reference: ISLHDQA242). We compared inpatient *C. difficile* acquisition rates over two equal periods (each spanning 996 days): the pre-COVID-19 period (September 16, 2017, to June 8, 2020) and the COVID-19 period (June 9, 2020, to March 1, 2023). *C. difficile* positivity was verified using our institutional Infection Management records, supplemented by chart review. This information was used to calculate *C. difficile* incidence rates (per 1,000 admissions and patient-days). Toxigenic *C. difficile* testing was routinely performed on diarrheal samples, excluding those with a negative result in the past seven days or a positive result within four weeks. Initial screening used the TECHLAB® C. DIFF QUIK CHEK COMPLETE™ to detect glutamate dehydrogenase (GDH) antigen and Toxins A and B. If GDH was positive but toxin negative, further testing with the Xpert® *C. difficile* PCR assay was conducted to detect toxin B, binary toxin, and the tcdC117 deletion found in Ribotype 027. A positive PCR result indicated *C. difficile* toxin gene detection. SARS-CoV-2 was detected using nasopharyngeal or throat swabs processed with various molecular PCR assays, including GeneXpert®, BD Max®, and Roche Cobas® Liat, introduced progressively to meet testing demands.

Inclusion criteria 

All adult patients (≥18 years old) who had diarrhea and tested positive for *C. difficile* at Wollongong Hospital during the specified study periods.

Exclusion criteria

Patients were excluded if they were under 18 years of age, did not have diarrhea, tested negative for *C. difficile*, or had a previous positive test within the preceding eight weeks. Additionally, patients with diarrhea who tested positive for *C. difficile* as well as another enteric pathogen (e.g., *Salmonella*, *Campylobacter*) were also excluded from the study.

Data collection

Data were extracted from electronic medical records, including patient demographics (age, sex), comorbidities (diabetes mellitus, chronic kidney disease, chronic obstructive pulmonary disease, malignancy, organ transplantation, inflammatory bowel disease), and clinical risk factors (immunosuppressive therapy, recent major surgery, gastrointestinal procedures, nasogastric tube use, and acid-suppressive therapy within 30 days). Additional variables comprised antimicrobial exposure (type and duration of antibiotics), COVID-19 status, hospitalization details (admission and discharge dates, ICU admission, CDI acquisition timing and severity), and CDI treatment regimens. Hospital activity data (total admissions, patient-days, ICU LOS) were collected, and hand hygiene compliance rates were obtained from infection control surveillance. Hand hygiene compliance was evaluated through direct observation by trained staff using the World Health Organization (WHO) "Five Moments" framework, with compliance calculated as correct actions per observed opportunities. The hospital’s hand hygiene protocol remained unchanged throughout the study.

Definitions

CDI was defined as diarrhea (≥3 loose stools in a 24-hour period) with one of the following criteria: a positive laboratory assay for *C. difficile* toxin A and/or B, or detection of a toxin-producing *C. difficile* organism in the stool by culture or other validated methods [22].

Healthcare-associated hospital-onset CDI was defined as symptom onset occurring more than 48 hours after admission to a healthcare facility [23].

Healthcare-associated community-onset CDI was defined as symptom onset occurring in the community or within 48 hours of hospital admission [23].

Severe CDI was defined as the presence of at least one of the following criteria: admission to the ICU due to CDI-related complications; identification of pseudomembranes on colonoscopy (if performed); radiological evidence of ileus, toxic megacolon, or pancolitis on abdominal imaging such as a computed tomography scan; surgical intervention related to CDI, such as colectomy; or the presence of clinical and laboratory indicators including a fever exceeding 38.5°C and a white blood cell (WBC) count greater than 20 × 10⁹/L [22].

Recurrence of CDI was defined as the reappearance of symptoms and a positive test for *C. difficile* within eight weeks of a previous episode, provided that the initial episode had resolved following appropriate treatment [22].

CDI incidence was calculated per 1,000 patient-days and per 1,000 patient admissions [24]:

$\text{CDI incidence}=\frac{\text{Number of CDI cases}}{\text{Total patient-days or admissions}} &times; 1000$

Statistical analysis

Data were analyzed using IBM SPSS Statistics for Windows, Version 20 (Released 2011; IBM Corp., Armonk, New York). Categorical variables were presented as counts and percentages, while continuous variables were summarized using means, standard deviations, medians, interquartile ranges (IQR), and ranges. Normality was assessed using the Shapiro-Wilk test. Group comparisons were made using the chi-square test for categorical variables; Fisher’s Exact test or Monte Carlo correction was applied when over 20% of cells had expected counts <5. For normally distributed continuous variables, Student’s t-test was used. Statistical significance was set at p < 0.05.

## Results

At Wollongong Hospital, 544 patients tested positive for CDI over the study period from September 16, 2017, to March 1, 2023. The electronic medical records of these patients were retrospectively reviewed by the research team.

In the pre-COVID-19 period (Group I), there were 215 cases of CDI among 142,620 admissions. Of these, 118 patients (54.9%) were identified as having healthcare-associated hospital-onset CDI, while 97 patients (45.1%) had healthcare-associated community-onset CDI. In the COVID-19 period (Group II), 329 cases of CDI were identified among 140,900 admissions. Of these, 173 patients (52.6%) were classified as having healthcare-associated hospital-onset CDI, and 156 patients (47.4%) had healthcare-associated community-onset CDI.

The demographic characteristics of the two groups, significant predisposing factors, and hand hygiene compliance rates are summarized in Table 1. There were no significant differences between the groups with regard to basic characteristics and predisposing factors, except for non-surgical gastrointestinal (GIT) procedures, which showed a notable difference. Overall hand hygiene compliance rates were similar between the two groups. However, when stratified by location, compliance rates were lower in the general wards during the COVID-19 period.

**Table 1:** Comparison of demographic characteristics, significant predisposing factors for CDI acquisition, and hand hygiene compliance rates between the two groups. *Nonsurgical. N: number, SD: standard deviation, DM: diabetes mellitus, CKD: chronic kidney disease, COPD: chronic obstructive pulmonary disease, IBD: inflammatory bowel disease, CDI: *Clostridioides difficile* infection, GIT: gastrointestinal tract, NGT: nasogastric tube, ICU: intensive care unit.

Demographic data	Group I (pre-COVID-19) (N = 215)	Group II (COVID-19) (N = 329)	P-value
Gender			
Male, n (%)	103 (47.9%)	133 (40.4%)	0.085
Female, n (%)	112 (52.1%)	196 (59.6%)	
Age (years), mean ± SD	66.32 ± 17.31	67.92 ± 17.34	0.245
Predisposing factors			
DM, n (%)	64 (29.8%)	85 (25.8%)	0.315
CKD, n (%)	49 (22.8%)	55 (16.7%)	0.078
COPD, n (%)	33 (15.3%)	51 (15.5%)	0.962
Malignancy, n (%)	93 (43.3%)	137 (41.6%)	0.709
Transplant, n (%)	10 (4.7%)	12 (3.7%)	0.566
IBD, n (%)	12 (5.6%)	16 (4.9%)	0.711
Immunosuppressant, n (%)	72 (33.5%)	123 (37.4%)	0.354
Recent major surgery prior to CDI, n (%)	59 (27.4%)	104 (31.7%)	0.530
GIT procedures*, n (%)	40 (18.6%)	36 (10.9%)	0.019
NGT insertion, n (%)	21 (9.8%)	35 (10.6%)	0.744
Ulcer prophylaxis, n (%)	132 (61.4%)	207 (62.9%)	0.720
Hand hygiene compliance rate %			
Wards correct/total moments (%)	49,602/56,051 (88.5%)	45,577/51,846 (87.9%)	0.003
ICU correct/total moments (%)	2,282/2,732 (83.5%)	1,930/2,301 (83.9%)	0.739
Overall correct/total moments in wards and ICU (%)	51,884/58,783 (88.3%)	47,507/54,147 (87.7%)	0.254

Not all CDI patients had received antibiotics prior to infection, 73% in the pre-COVID-19 period and 76% during the COVID-19 period, with no statistically significant difference between the groups. Among those who received antibiotics, the mean duration of antibiotic use was similar in both groups. Notably, macrolides were used more frequently in the pre-COVID-19 period, while aminoglycosides were more commonly administered in the COVID-19 period prior to CDI acquisition. Table 2 summarizes antibiotic usage, duration of exposure prior to CDI onset, and the different classes of antibiotics administered.

**Table 2:** Comparison of antibiotic usage, duration before CDI acquisition, and antibiotic classes between study groups. CDI: *Clostridioides difficile* infection, N: number, SD: standard deviation, Min: minimum, Max: maximum, IQR: interquartile range.

Antibiotic exposure prior to CDI	Group I (pre-COVID-19) (N = 215)	Group II (COVID-19) (N = 329)	p-value
Number of patients who received antibiotics prior to CDI			
Number of CDI patients, n (%)	157 (73%)	250 (76%)	0.436
Duration of antibiotics prior to CDI (days)			
Min–Max	1.0–83.0	1.0–113.0	0.458
Mean ± SD	8.0 ± 9.97	8.17 ± 9.68	
Median (IQR)	6.0 (2.0–8.0)	6.0 (3.0–12.0)	
Antibiotic classes used prior to CDI acquisition			
Penicillins, n (%)	88 (40.9%)	161 (48.9%)	0.067
Cephalosporins, n (%)	107 (49.8%)	146 (44.4%)	0.218
Macrolides, n (%)	14 (6.5%)	9 (2.7%)	0.032
Fluoroquinolones, n (%)	7 (3.3%)	8 (2.4%)	0.570
Carbapenems, n (%)	14 (6.5%)	22 (6.7%)	0.936
Tetracyclines, n (%)	4 (1.9%)	14 (4.3%)	0.127
Glycopeptides, n (%)	39 (18.1%)	51 (15.5%)	0.418
Aminoglycosides, n (%)	32 (14.9%)	79 (24.0%)	0.010
Sulfonamides, n (%)	26 (12.1%)	28 (8.5%)	0.172
Nitroimidazole, n (%)	35 (16.3%)	59 (17.9%)	0.618
Lincosamides, n (%)	3 (1.4%)	10 (3.0%)	0.220

There were no significant differences between the study groups in terms of CDI type, time to acquisition, severity, need for ICU admission, ICU and hospital length of stay, or mortality rate. However, during the COVID-19 period, the CDI recurrence rate, as well as the incidence per 1,000 patient admissions (2.2 vs. 1.67 cases) and per 1,000 patient-days (0.64 vs. 0.45 cases), was higher compared to the pre-COVID-19 period. These outcome parameters, along with the antibiotics used to treat CDI, are presented in Table 3.

**Table 3:** Comparison of different outcome parameters and therapeutic antibiotics for CDI between study groups. *Fidaxomicin, fecal matter transplant (FMT). **Oral vancomycin plus metronidazole. HCA: healthcare-associated, N: number, SD: standard deviation, Min: minimum, Max: maximum, IQR: interquartile range, CDI: *Clostridioides difficile* infection, ICU: intensive care unit, LOS: length of stay.

Outcome parameters	Group I (pre-COVID-19) (N= 215)	Group II (COVID-19) (N = 329)	p-value
Acquisition type			
HCA-hospital-onset, n (%)	118 (54.9%)	173 (52.6%)	0.599
HCA-community-onset, n (%)	97 (45.1%)	156 (47.4%)	
Time to acquisition (Days)			
Min–Max	0.0–105.0	0.0–134.0	0.388
Mean ± SD	7.64 ± 13.66	6.68 ± 11.95	
Median (IQR)	3.0 (1.0–9.0)	3.0 (1.0–8.0)	
Severity of CDI			
Severe CDI, n (%)	37 (17.2%)	64 (19.5%)	0.511
Recurrence of CDI			
Recurrent CDI, n (%)	12 (5.6%)	36 (10.9%)	0.031
Need for ICU admission			
CDI cases needed ICU admission, n (%)	39 (18.1%)	67 (20.4%)	0.522
ICU LOS (Days)			
Min–Max	1.0–16.0	0.125–22.0	0.415
Mean ± SD	5.08 ± 4.29	5.79 ± 4.68	
Median (IQR)	4.0 (2.0–8.0)	4.0 (2.0–8.0)	
Hospital LOS (Days)			
Min–Max	0.0–194.0	0.0–200.0	0.788
Mean ± SD	19.16 ± 22.67	17.90 ± 21.12	
Median (IQR)	12.0 (13.0–47.0)	12.0 (14.0–38.0)	
Mortality			
Died, n (%)	20 (9.3%)	29 (8.8%)	0.846
CDI incidence per 1,000 patient-days			
Total CDI cases/1,000 patient-days, n/1,000 (%)	209/467,384 (0.45)	310/487,715 (0.64)	<0.001
Community-onset, n/1,000 (%)	91/467,384 (0.2)	137/487,715 (0.28)	0.006
Hospital-onset, n/1,000 (%)	118/467,384 (0.25)	173/487,715 (0.36)	0.004
CDI incidence per 1,000 patient admissions			
Total CDI cases/1,000 patient admissions, n/1,000 (N) (%)	209/142,620 (1.47)	310/140,900 (2.2)	<0.001
Community-onset, n/1,000 (%)	91/142,620 (0.64)	137/140,900 (0.97)	0.002
Hospital-onset, n/1,000 (%)	118/142,620 (0.83)	173/140,900 (1.23)	0.001
CDI incidence among COVID-19-positive patients per 1,000 COVID-19 admissions			
CDI cases in COVID-19-positive patients/1,000 COVID-19 admissions, n/1,000 (%)	0/17 (0)	22/3,893 (5.65)	1
Antibiotics used to treat CDI			
Oral vancomycin, n (%)	76 (35.3%)	192 (58.4%)	<0.001
Metronidazole, n (%)	94 (43.7%)	41 (12.5%)	>0.001
Others* or combination**, n (%)	39 (18.1%)	71 (21.6%)	0.329

In terms of location at the time of CDI diagnosis, 491 out of 544 patients (90.3%) were in a ward setting, 196 during the pre-COVID-19 period and 295 during the COVID-19 period. The remaining 53 patients (9.7%) were in the ICU, 19 in the pre-COVID-19 period and 34 in the COVID-19 period. There were no statistically significant differences between the two groups in terms of diagnosis location.

**Figure 1:**
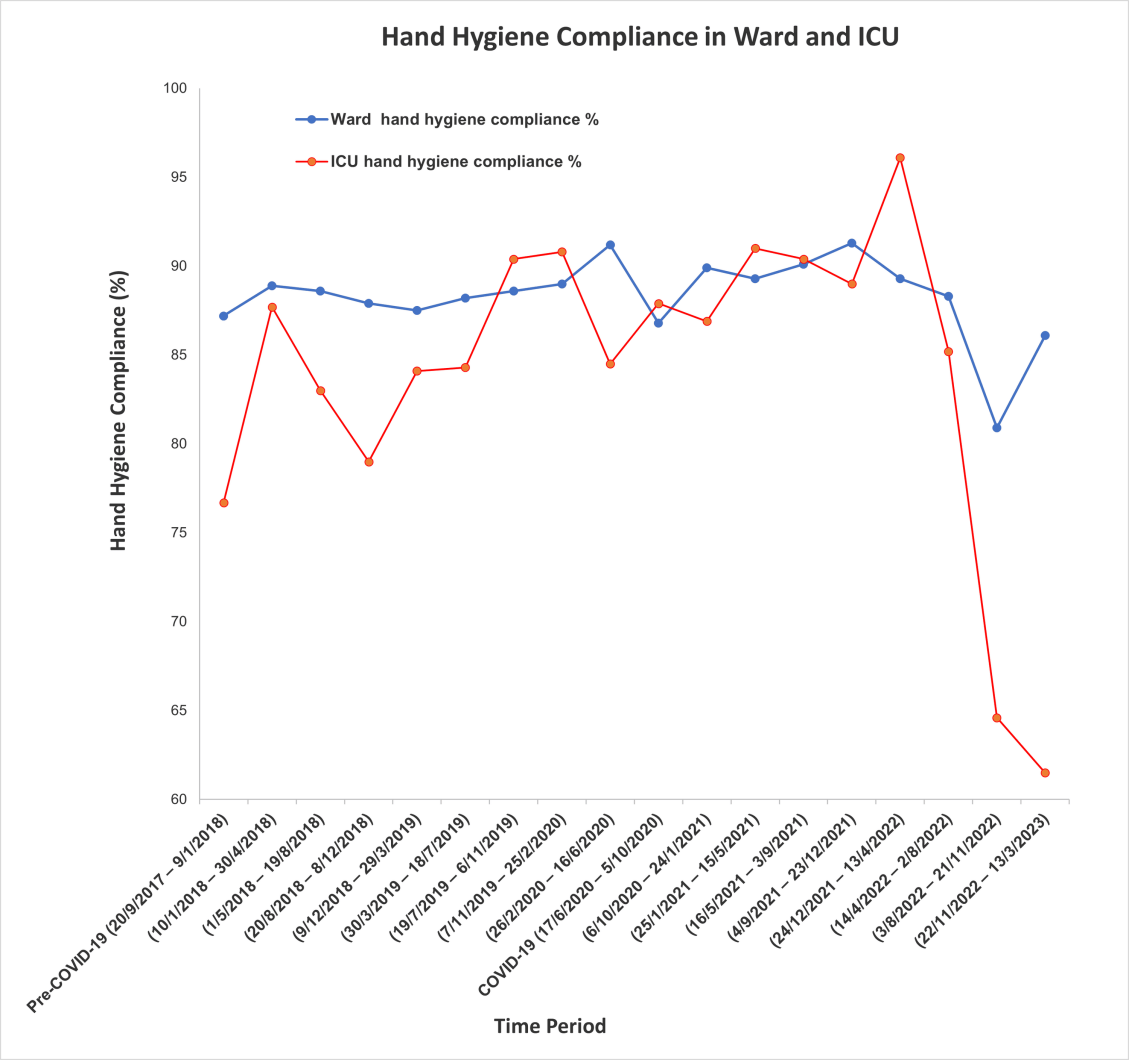
Comparison of hand hygiene compliance between ICU and general ward settings.

Hand hygiene compliance rates varied across the study periods. Overall, general wards demonstrated better compliance than the ICU. When comparing the two periods, compliance in the general wards decreased slightly but significantly during the COVID-19 period (from 88.5% to 87.9%, p = 0.003). Although the absolute change was small (0.6%), the large sample size likely contributed to the statistical significance. In contrast, ICU compliance remained stable (83.5% to 83.9%, p = 0.739). Figure 1 illustrates the hand hygiene compliance rates in both general wards and ICU across the study periods, based on infection control data.

A total of 49 patients (9%) did not survive. When comparing non-survivors to survivors, the severity of CDI was higher in the COVID-19 group, and the need for ICU admission was greater in both periods. Table 4 compares non-survivors to survivors regarding CDI severity, recurrence, and the need for ICU admission in both periods.

**Table 4:** Comparison of CDI severity, recurrence rates, and ICU admission requirements between survivors and non-survivors in both groups. CDI: *Clostridioides difficile *infection, ICU: Intensive Care Unit.

CDI complications	Group I (pre-COVID-19) (N=215)			Group II (COVID-19) (N=329)		
	Survived (N=195 (90.7%))	Non-survived (N=20 (9.3%))	p-value	Survived (N = 300 (91.2%))	Non-survived (N=29 (8.8%))	p-value
Patients who had severe CDI, n (%)	31 (15.9%)	6 (30%)	0.12	54 (18%)	10 (34.5%)	0.032
Patients who required ICU, n (%)	31 (15.9%)	8 (40%)	0.014	56 (18.7%)	11 (37.9%)	0.014
CDI recurrence, n (%)	10 (5.1%)	2 (10%)	0.309	35 (11.7%)	1 (3.4%)	0.227

A total of 22 patients (4%) had co-infections with both COVID-19 and CDI. Analysis of this subgroup revealed no significant differences in mortality, hospital LOS, need for ICU admission, CDI severity, or recurrence compared to COVID-19 negative patients. Table 5 compares COVID-19 negative and COVID-19 positive patients in terms of CDI severity, recurrence, need for ICU admission, hospital LOS, and mortality rate.

**Table 5:** Impact of COVID-19 co-infection on clinical outcomes in CDI-positive patients. CDI: *Clostridioides difficile* infection, ICU: Intensive Care Unit, LOS: length of stay, SD: standard deviation, Min: minimum, Max: maximum, IQR: interquartile range.

Parameters	CDI-positive patients		p-value
	COVID-19-negative (N = 522 (95.96%))	COVID-19-positive (N = 22 (4.04%))	
Severity of CDI, n (%)	97 (18.6%)	4 (18.2%)	1.000
Need for ICU admission, n (%)	104 (19.9%)	2 (9.1%)	0.278
Recurrence, n (%)	45 (8.6%)	3 (13.6%)	0.431
Hospital LOS in days			
Min–Max	0.0 - 200.0	0.0 - 61.0	0.053
Mean ± SD	18.22 ± 21.91	22.64 ± 16.88	
Median (IQR)	12.0 (5.0 - 23.0)	17.50 (8.0 - 30.0)	
Mortality rate			
Died, N (%)	47 (9.0%)	2 (9.1%)	1.000

## Discussion

In September 2020, Wollongong Hospital was designated as a COVID-19 center. Following an increase in COVID-19 admissions, a concurrent rise in CDI cases was observed. To investigate this trend, we conducted a retrospective study comparing the incidence of CDI during the COVID-19 period with a matched pre-COVID-19 control period.

During the COVID-19 period, the incidence of CDI, both community- and hospital-onset, was significantly higher compared to the pre-COVID-19 period, rising from 0.45 to 0.64 cases per 1,000 patient-days (p < 0.001). Similarly, incidence per 1,000 admissions increased from 1.47 to 2.2 cases (p < 0.001), indicating a heightened burden of CDI during the pandemic. This trend aligns with findings from the Australian Commission on Safety and Quality in Health Care, which reported a 29% increase in CDI separations between 2020 and 2021. Nationally, community-onset CDI accounted for over 80% of all cases, compared to 46.5% in our study, while hospital-onset CDI represented less than 20% nationally versus 53.5% in our data [25]. It is worth noting that our study did not include outpatient populations, which may explain this discrepancy.

Similarly, several international studies have reported a higher incidence rate of CDI during COVID-19. Kwon et al. [6] observed a higher rate of hospital-onset CDI during the COVID-19 pandemic in a tertiary care hospital in Missouri. Another retrospective observational study conducted in a tertiary hospital in Greece between May 2021 and October 2022 also found an increased prevalence of CDI during the pandemic [7]. Furthermore, in Brazil, a retrospective study found that CDI incidence among ICU patients doubled during the COVID-19 period compared to the pre-COVID-19 period [26]. Likewise, a retrospective study from Poland reported a higher CDI incidence during the COVID-19 period [5]. In contrast to these findings, and to our results, studies from Ireland, Romania, the United Kingdom, the United States, and Spain found no change or even a decrease in CDI incidence during the pandemic [8,11,16,27-30].

Hand hygiene is a cornerstone of infection prevention, particularly in controlling the spread of *C. difficile *in healthcare settings. Unlike many pathogens, *C. difficile* forms spores that are resistant to alcohol-based hand sanitizers, making handwashing with soap and water the recommended method for effectively removing these spores from hands. The World Health Organization emphasizes the importance of this practice, especially after contact with patients infected with *C. difficile*, to prevent transmission [31]. In our study, we observed a statistically significant decrease in hand hygiene compliance during the COVID-19 period compared to the pre-COVID-19 period (87.9% vs. 88.5%, p = 0.003). This decline in hand hygiene compliance may have contributed to the increased incidence of CDI observed during the COVID-19 period. Nonetheless, compliance in our institution remained above the international benchmark of 80% and exceeded the national average compliance rate of 86.8%, as reported in the latest National Hand Hygiene Dashboard (Period 3, 2024), which included data from 1,023 public and private healthcare organizations across Australia [32].

Antibiotic use is a well-established risk factor for CDI acquisition, particularly with agents such as cephalosporins, carbapenems, fluoroquinolones, and clindamycin [33]. In our study, there was no significant difference in overall antibiotic use or in the duration of antibiotic exposure prior to CDI onset between the pre-COVID-19 and COVID-19 periods. However, aminoglycoside use was significantly higher during the COVID-19 period compared to the pre-COVID-19 period (24% vs. 15%, p = 0.01). Aminoglycosides are generally considered to carry a lower risk of contributing to CDI compared to higher-risk antibiotic classes such as clindamycin and cephalosporins [34].

Of the 544 patients, 101 (18.5%) had severe CDI, with no significant difference in severity between the pre-COVID-19 and COVID-19 periods. However, severe CDI and the need for ICU admission were more frequent among patients who did not survive in both study periods. Some studies have suggested that COVID-19 may contribute to increased CDI severity [35-38]; however, this trend was not observed in our cohort.

We observed a significantly higher rate of CDI recurrence during the COVID-19 period compared to the pre-COVID-19 period (10.9% vs. 5.6%, p = 0.03). Despite this increase, the overall recurrence rate in our study remains lower than previously reported Australian rates, which range from 25% to 47% [25]. The near doubling of recurrence during the COVID-19 period suggests a potential association between the pandemic and increased CDI recurrence. Although chronic kidney disease, steroid use, and proton pump inhibitor (PPI) use are known risk factors for recurrence [39], no significant differences in these factors were identified between the two study periods. Additionally, CDI recurrence is more common with vancomycin treatment compared to fidaxomicin [40]. In our study, the use of oral vancomycin was significantly higher during the COVID-19 period (58.4% vs. 35.3%, p < 0.001), which may have contributed to the increased recurrence rate.

Among the 22 patients with co-infections of CDI and COVID-19, we found no significant differences compared to patients with CDI alone in terms of CDI severity, ICU admission, recurrence rate, hospital length of stay, or mortality. However, other studies have reported contrasting outcomes. Stoian et al. [41] observed a significant increase in ICU length of stay and mortality among co-infected ICU patients. Similarly, Awan et al. [38] reported worse outcomes in co-infected patients, including higher mortality, morbidity, and longer hospital stays. Cerbulescu et al. [36] also found that co-infected patients had increased ICU admissions, longer durations of mechanical ventilation, and higher mortality rates.

This study has several limitations. First, it was conducted at a single center, which may limit the generalizability of the findings to other healthcare settings with different patient populations, resources, or infection control practices. Second, the retrospective design is inherently subject to information and selection bias, as it relies on the accuracy and completeness of existing medical records. Additionally, unmeasured confounders, including undocumented outpatient antibiotic use (type and duration), loss to follow-up from inter-hospital transfers, and infection control variables (PPE adherence, environmental cleaning protocols, staffing challenges), may influence outcome interpretation, though not our primary objective of determining CDI rates. These limitations underscore the need for multicenter prospective studies to assess causality.

## Conclusions

The impact of the COVID-19 pandemic on CDI acquisition remains inconclusive, with existing literature reporting mixed findings, ranging from no effect to both increased and decreased incidence. In our study, we observed a significant rise in CDI incidence during the COVID-19 period compared to the pre-COVID-19 period. However, this increase was not associated with a corresponding rise in overall hospital length of stay or mortality. These findings highlight the need for ongoing surveillance and targeted infection control strategies, particularly during periods of healthcare system strain such as pandemics.
